# Supplementary material for: Effectiveness of Hospital Fit on Physical Activity in Hospitalized Patients: A Stepped-Wedge Cluster-Randomized Trial and Process Evaluation
Source: Sensors (Basel). 2024 Sep 12;24(18):5920. doi: 10.3390/s24185920 (PMC11435518; doi:10.3390/s24185920)
Supplement: Supplementary file 1 [file sensors-24-05920-s001.zip › sensors-3168588-supplementary.pdf]

Supplementary Materials

Supplementary File S1 – Patient-reported questionnaire

General information

Study number

……

Date

…………… (day) - …………… (month) - …………… (year)

Use of Hospital Fit

I have used Hospital Fit today

- Yes
- No

If yes: I have used Hospital Fit ……… times today

I have used the following functionalities:

- Physical activity overview

If applicable, I have used the physical activity overview ……… times today

- Recovery overview

If applicable, I have used the recovery overview ……… times today

- Patient specific exercise program

If applicable, I have used the patient specific exercise program ……… times today

- Information on physical activity

If applicable, I have used the information on physical activity ……… times today

- Reminder function

Supplementary File S2- - Interview guide

Patients

Introduction

I would like to start the interview. We will also start immediately with a sound recording, so that the sound of our conversation will be recorded. Do you agree with this sound recording?

I would like to ask you to answer as much as possible during the interview. This will give me a picture of your thoughts and experiences. It helps tremendously if you give examples of situations and think out loud. There are no wrong answers. You can stop the interview at any time. In addition, you don't have to answer questions you don't want to answer. Do you have any questions at this time?

Effectiveness of Hospital Fit

I'll start with a few open-ended questions about your thoughts and experiences regarding Hospital Fit. We will talk about the activity tracker and the smartphone application

**Experiences with Hospital Fit**	What is it like for you to have a movement meter on? And what is it like for you to use the smartphone application?<br>Probes: Attaching activity tracker, wearing activity tracker, installing application, opening application, syncing, what helped, what didn't help
**Perceived effectiveness**	How has the activity tracker and smartphone application affected your movement? How has the activity tracker and smartphone application influenced your movement?<br>Probes: Effect, health, recovery
**Knowledge and skills**	What knowledge do you need to use the activity tracker and smartphone application?<br>And what skills? What skills do you need to be able to use the activity tracker and smartphone application?<br>Probes: Information, training, support by healthcare professionals
**Attitude**	Did the activity tracker and smartphone application affect your attitude toward exercise during hospitalization? Can you share a little more about this? Have you changed your thinking about exercise during hospitalization because of the activity tracker and smartphone application?<br>Probes: Importance of exercise, monitoring exercise, support
**Behavior**	What (daily) activities do you perform? What do you do during hospitalization? Do you do anything different now because of the activity tracker and smartphone application?<br>Probes: Lying, sitting, walking, climbing stairs, going to toilet, washing, showering, cycling, exercising with close-relatives
**Implementation and improvement**	Are you able to use the activity tracker and smartphone application? Do you have any wishes for improvement? What could be improved that would make you use the activity tracker and smartphone application more?<br>Probes: Manual, counseling by therapist, involvement of close-relatives
**Maintenance**	Would you use the activity tracker and smartphone application long-term during a hospital stay? Why and why not?<br>Probes: Benefits, barriers

Closure

Now let's move on to the closing. Anything you would like to share about your thoughts and experiences about the activity tracker and smartphone application? Is there anything that happened that hasn't already come up during the interview? Do you have any questions of your own? Would

you like to stay informed about the results of this research? This concludes the interview and we will stop the audio recording.

Many thanks for your participation.

Please note that the interview guide is translated from Dutch to English with DeepL.com

Healthcare professionals

Introduction

Welcome to this focus group meeting. My name is Niek Koenders and I am the chair of the conversation. This talk is held as part of the study "Effectiveness of Hospital Fit. Over the past few months Hospital Fit - an activity tracker and smartphone application - has been deployed in the Cardiology department. We would like to evaluate how this has been for you.

Before we begin, I'd like to start with a brief round of introductions. Could you briefly state your name and position?

I would like to start the interview. We will also start immediately with an audio recording, so that the sound of our conversation is recorded. Are you okay with this sound recording?

I would like to ask you to answer as fully as possible during the interview. This will give me a picture of your thoughts and experiences. It helps tremendously if you give examples of situations and think out loud. There are no wrong answers. It is possible to respond to each other. Please, this is the intention! Please do so calmly and respectfully. Do you have any questions at this point?

Effectiveness of Hospital Fit 2.0

I'll start with a few open-ended questions about your thoughts and experiences regarding Hospital Fit. We will talk about the activity tracker and the smartphone application.

**Experiences with Hospital Fit**	What was your experience with Hospital Fit? What was it like to use the activity tracker? And what was it like to use the application?<br>Probes: Attachting activity tracker, wearing activity tracker, installation, opening, synchronising, what helped and what didn't help?
**Perceived effectiveness**	How do the activity tracker and smartphone application affect patient exercise?<br><br>Probes: Effect in subgroups, outcomes you want to learn from as a healthcare professional, what were important outcomes for you to determine effect?
**Knowledge and skills**	What knowledge do you need to use the activity tracker and smartphone application?<br>And what skills? What skills do you need to be able to use the activity tracker and smartphone application?<br>Probes: Information, training, patient knowledge and skills, support by healthcare professionals
**Attitude**	Did the activity tracker and smartphone application affect your attitude toward exercise during hospitalization? Can you share a little more about this? Have you changed your thinking about exercise during hospitalization because of the activity tracker and smartphone application?<br>Probes: Importance of exercise, monitoring exercise, support
**Behavior**	How did Hospital Fit affect your work as a healthcare professional? Do you do anything differently now because of the activity tracker and smartphone application?<br>Probes: Diagnosing, advising, treating, evaluating
**Implementation and improvement**	Do you manage to use the activity tracker and smartphone application? What could be improved that would make you use the activity tracker and smartphone application more?

**Maintenance**	Probes: Researcher presentation, researcher helpline, manuals, therapist guidance<br>Would you use the activity tracker and smartphone application long-term? Why and why not? What do you need for long-term use?<br>Probes: Benefits, barriers

Closure

Now let's move on to the closing. Anything you would like to share about your thoughts and experiences about the activity tracker and smartphone application? Is there anything that happened that hasn't already come up during the interview? Do you have any questions of your own? Would you like to stay informed about the results of this research? This concludes the interview and we will stop the audio recording.

Many thanks for your participation.

Please note that the interview guide is translated from Dutch to English with DeepL.com

Supplementary File S3 – Details of the primary analysis

Regression analysis
Outcome: Time walking in minutes per day
Variable: Group (non-intervention versus intervention)
Random: Constant
Method: Enter

				95% confidence interval for B	
	**B**	**Standard error**	**p-value**	**Lower bound**	**Upper bound**
**Constant**	35	3	<0.001	20	50
**Group**	36	5	<0.001	25	47

Linear Mixed Model
Outcome: Time walking in minutes per day
Fixed factors: Group (non-intervention versus intervention)
Random: Intercept
Subject: Cluster

				95% confidence interval for B	
	**B**	**Standard error**	**p-value**	**Lower bound**	**Upper bound**
**Intercept**	35	3	0,008	21	48
**Group**	37	5	<0.001	27	46

Linear Mixed Model
Outcome: Time walking in minutes per day
Fixed factors: Group (non-intervention versus intervention) and Step (month 1-8)
Random: Intercept
Subject: Cluster

				95% confidence interval for B	
	**B**	**Standard error**	**p-value**	**Lower bound**	**Upper bound**
**Intercept**	26	6	0.004	11	40
**Group**	20	11	0.075	-2	41
**Step**	4	2	0.083	0	8
